# Fertility Enhancement but Premature Ovarian Failure in *esr1*-Deficient Female Zebrafish

**DOI:** 10.3389/fendo.2018.00567

**Published:** 2018-09-24

**Authors:** Yu Chen, Haipei Tang, Le Wang, Jianan He, Yin Guo, Yun Liu, Xiaochun Liu, Haoran Lin

**Affiliations:** State Key Laboratory of Biocontrol, Institute of Aquatic Economic Animals and Guangdong Province Key Laboratory for Aquatic Economic Animals, School of Life Sciences, Sun Yat-Sen University, Guangzhou, China

**Keywords:** *esr1*, fertility, POF, mTOR pathway, zebrafish

## Abstract

It is well established that estrogens regulate female reproduction through estrogen receptors (ERs) in the ovary. However, the precise physiological role of estrogen/ER signaling in reproduction processes remains poorly defined in zebrafish. In this study, we successfully generated an ERα (*esr1*) mutant line in zebrafish via transcription activator-like effectors nucleases (TALENs). It was found in the mutant females that the fertility was enhanced and the ovarian histology was normal at 90 days post-fertilization (dpf). However, the number of fertile females decreased with age. By 180 dpf, *esr1* mutant females were infertile with degenerated ovaries, while the age-matched wild-type females were still fertile. Additionally, few large vitellogenic granules can be found in full grown (FG) follicles at 90 dpf and the expression of *vtg* genes were down-regulated at both 90 and 180 dpf in *esr1* mutant zebrafish. Moreover, steroidogenesis pathway and mTOR signaling pathway were over-activated at 90 dpf, but declined prematurely in *esr1* mutant zebrafish by 180 dpf. Collectively, the present study provides evidence that *esr1* is fundamental for ovarian maintenance in zebrafish.

## Introduction

Estrogens play key roles in ovarian maintenance in vertebrates (1). The biological functions of estrogens are exerted via the estrogen receptors (ERs), a series of ligand-dependent nuclear transcription factors. In vertebrates, there are two cognate subtypes of nuclear ER, estrogen receptor alpha (ERα) and estrogen receptor beta (ERβ), the ligand-activated transcription factors (2), and one subtype of membrane ER, GPER, a G-protein coupled receptor (3). Classically, the genomic actions are primarily mediated by nuclear ERs, while the non-genomic effect was mediated by membrane-associated ERs (4). In mammals, the two subtypes of nuclear ER exhibit differential or overlapping tissue distribution as well as remarkable differences in ligand-binding and transcriptional properties (5). Traditionally, ERβ is considered to be the predominant estrogen receptor form in ovary (6, 7). It is essential for ovulation efficiency and embryogenesis rather than sexual differentiation and fertility (8–11). However, genetic disruption of ERβ (*Esr2*) in mice did not cause severe fertility defects (8, 12). On the other hand, accumulating evidence has pointed out that ERα is a critical factor in ovarian development and functional maintenance.

In human, defects of the ERα encoding gene, *ESR1*, were reported to be closely related to premature ovarian failure (POF) syndrome (13–17), an ovarian defect characterized by premature depletion of ovarian follicles or arrested folliculogenesis (18). Genetic evidences were further provided by mammalian models that different lines of *Esr1* knockout mice displayed diverse degrees of fertility defects in adulthood with severe ovarian disorders. Global *Esr1* knockout female mice were generally considered to be infertile in adulthood, which is similar to acyclic adult animals (12, 19–22). By 10 days age, the ovaries of *Esr1* null mice were indistinguishable with that in wild-type ones, containing histologically normal primordial and primary follicles. However, functional maturation of pre-ovulatory follicles in 20–50 days age was arrested, resulting in atresia or anovulatory follicles, which formed large, hemorrhagic cysts in many cases. In addition, corpora lutea were absent, indicating that the biochemical and mechanical processes for ovulation were compromised in global *Esr1* knockout mice (12). Furthermore, both neuron-specific and pituitary-specific deletion of *Esr1* impaired the feedback regulation on gonadotropin by estrogen, which consequently impaired the fertility of female mice (23, 24). Interestingly, female mice with specific deletion of *Esr1* gene in ovarian theca cells were fertile and cycling until 4-month old. But thereafter they began to display an erratic pattern of estrous cycles and finally lost fertility prematurely by the age of 6 months (7). In light of these, there is sufficient evidence to conclude that ERα plays an important role in the process of female reproduction in mammals.

ERα is also deeply involved in diverse reproductive processes in teleosts. It comprehensively elevated in hypothalamus-pituitary-gonadal (HPG) axis during the sexual maturation period in female eels (25). In addition, it was reported in many teleost species that ERα tightly coupled to up-regulation of vitellogenins upon estrogen stimulation (25–28). More than one alpha subtype of estrogen receptors were identified in teleost due to the genome duplication, but one of these duplicated ERαs has been lost in many fish species (29). In zebrafish, ERα is the only one alpha subtype of estrogen receptor (30). Similar to mammals, ERα enriched in the follicle layer in the ovary of zebrafish. It increased at pre-vitellogenic stage and later peaks at mid-vitellogenic stage follicles temporally (31). Furthermore, ERα is an essential factor widespread in HPG axis and reproduction system of zebrafish. Our recent study has confirmed that *esr1* was colocalized with *lhb* in pituitary and mediates the direct estrogenic effects on *lhb* in pituitary (32). Taken together, lines of evidence suggests that ERα is located comprehensively in reproduction system of teleosts and deeply involved in regulation of various reproductive processes.

However, the role of ERα in ovary development and maintenance has not yet been completely clarified. In this study, we generated an ERα (*esr1*) mutant zebrafish line via TALENs and evaluated the fertility and ovarian histology of the females. It was found that *esr1* deficient zebrafish developed enhanced fertility with normal ovarian histology at 90 dpf, but developed POF with degenerated ovaries at 180 dpf. As well, defect in vitellogenesis was found in these zebrafish. Further investigation showed that the alteration of steroidogenesis pathway and mTOR signaling pathway may be relevant to the reproduction disorder in ERα deficient zebrafish.

## Materials and methods

### Zebrafish husbandry

AB strain zebrafish (*Danio rerio*) were maintained in a 14 h light (8:00–22:00): 10 h dark (22:00–8:00) cycle at 28 ± 1°C in our laboratory in the Sun Yat-Sen University, following the protocols described in *The zebrafish book: a guide for the laboratory use of zebrafish* (33). The wild-type and *esr1* mutant zebrafish were raised separately in tanks with the same size in the same system. About 35 zebrafish were maintained in each tank containing 10 L water. The larvae and adult zebrafish were fed with brine shrimp (hatched from eggs in 30 mL in 8 L saline water) twice daily. All animal experiments conducted were in accordance with the guidelines and approval of the respective Animal Research and Ethics Committees of Sun Yat-Sen University.

### Establishment of *esr1* gene disruption zebrafish line via TALENs

**Figure 1:**
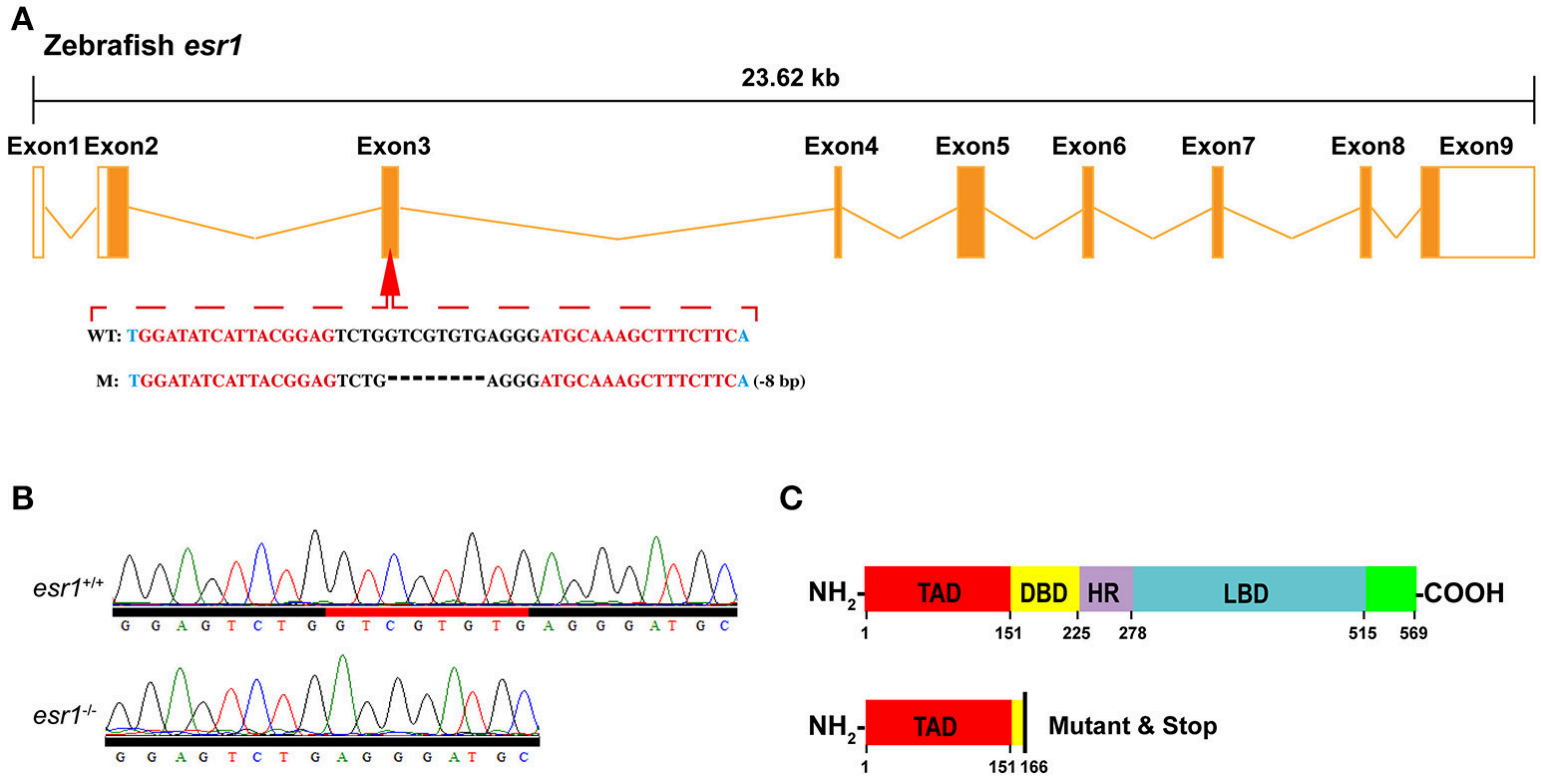
Targeted disruption of zebrafish *esr1* gene. **(A)** Schematic representation of genomic structure of zebrafish *esr1* gene and the TALENs target sites on zebrafish *esr1* gene. **(B)** Sequencing results of *esr1* gene from wild-type (*esr1*^+/+^) and homozygous (*esr1*^−/−^) zebrafish. **(C)** Mutation in exon 3 of *esr1* causes early termination of translation. TAD, transactivation domain; DBD, DNA-binding domain; HD, hinge domain; LBD, ligand binding domain.

To obtain the zebrafish mutant line, specific TALEN target sites were designed locating on the third exon (Figure 1A) of zebrafish *esr1* (Gene ID: 259252), which encodes the DNA-binding domain (Figure 1C). Paired TALENs were constructed with the Golden Gate TALEN Kit (Addgene, Cambridge, MA, USA) as reported (34, 35). Approximately 300 pg TALEN mRNAs were microinjected into one-cell stage zebrafish embryos and then reared at the temperature of 28.5°C. Twenty four hours after injection, 10 embryos were collected for DNA extraction to check whether the targeted genomic fragment was deleted. The target genomic regions were amplified by PCR and subcloned into the pTZ57R/T vector (Fermentas). Single colonies were genotyped by sequencing.

To obtain germline mutations, the P0 generation zebrafish were raised to adulthood and mated to wild-type fish to generate heterozygous F1 offspring. The heterozygous F1 generation fish were genotyped via fin clip assay and the individuals with frame-shift sequence alterations were selected. Males and females of F1generation carrying the same mutation were mated to produce F2 homozygous mutants which were genotyped via PCR and sequencing. Specific primers for PCR genotyping are listed in Supplementary Table 1.

### Fertility, fecundity, and fertilization rate assessment

A tracing fertility assessment was conducted with 10 wild-type and 10 mutant female zebrafish for 3 months (from 90 to 180 days post-fertilization). The wild-type and mutant females were one-to-one paired with wild-type males every 5 days interval. The number of spawned individuals and non-spawned individuals were recorded for each batch and the spawning ratio was calculated as (number of spawned individuals/sum accessed) × 100%.

To induce spawning, one female was paired with one male in a spawning tray. One hour after light on in the morning, spawned eggs were collected and counted. Fecundity is the count of number of eggs spawned (36). The developing embryos were maintained in 30% Danieau's solution at 28°C. The number of fertilized and unfertilized eggs were identified with a dissecting microscope at 4 h post-fertilization (hpf). The fertilization ratio was calculated as (fertilized eggs/total eggs) × 100%. Data of fecundity and fertilization rate were obtained from 10 independent crosses.

### *In vivo* exposure experiment

For *in vivo* drug exposure, wild-type female zebrafish (*n* = 6) were exposed to 50 nM of rapamycin (MCE, USA) for 14 days. The water in each tank was replaced daily. After drug treatment, zebrafish were anesthetized and sacrificed. Body weight and gonad weight were measured and the ovaries were prepared for histological analyses.

### Morphological and histological analyses

Morphological and histological analyses were performed as described (36, 37). Briefly, morphology and histology of the zebrafish ovaries (*n* = 6) was examined at 60, 90, 120, and 180 days post-fertilization (dpf). Zebrafish were euthanized with MS-222 and images were taken with a digital camera. Body length and body weight were measured. Then the ovary was dissected for gonad weight measurement and histological examination. The gonad-somatic index (GSI) was calculated as (gonad weight/body weight) × 100%. For gonad histology analyses, the ovary samples were fixed in paraformaldehyde overnight at 4°C. The samples were dehydrated through a graded series of ethanol and embedded in paraffin and serially cut into 7 μm sections on a Leica microtome. After rehydration, the sections were stained in hematoxylin and eosin in turn and mounted with Canada balsam (Sigma-Aldrich, USA) for microscopic examination. The division of follicle stages was referred to previous works (38, 39).

### Whole-body steroid hormone determination

Whole-body steroid measurement was performed as described previously (37). Firstly, female zebrafish (*n* = 6) were anesthetized with MS-222. Body weight of each zebrafish was measured prior to homogenization. They were then homogenized in 3 mL of PBS in glass tubes. Five millilitre of diethyl ether was added to each tube, vortexed for 1 min, centrifuged at 3,000 g for 2 min, and then frozen in a methanol/dry ice bath. The ether layer was poured into another set of tubes and evaporate the diethyl ether to dryness in a fuming hood. This procedure was repeated twice to enhance the extraction efficiency and the final product was dissolved in PBS solution. The estradiol level was accessed with Estradiol ELISA Kit (Cayman Chemical Company, Ann Arbor, MI, USA) and 11-KT with 11-keto Testosterone ELISA Kit (Cayman Chemical Company, Ann Arbor, MI, USA). The assays were conducted following the manufacturer's instructions. The standard series and all samples were conducted triplicated.

### RNA isolation and RT-PCR

Total RNA was extracted from the ovary and liver samples (*n* = 6) with TRIzol reagent (Invitrogen, USA). As follow, 1 μg total RNA was used as template to produce cDNAs with Rever Tra Acea-first strand cDNA Synthesis Kit (TOYOBO, Japan). The transcriptional level of the target genes was measured by the ABI Real-Time PCR Fast System with SYBR Green PCR Master Mix Kit. Procedure of quantitative RT-PCR was shown: denaturation at 95°C for 10 min, followed by 40 cycles of 95°C for 15 s, 58°C for 15 s, 72°C for 20 s, and then 84°C for 10 s (fluorescent data collection). All mRNA quantification data were normalized to *ef1a* and presented as fold changes to relative control groups. The specific primers used in this study are listed in Supplemental Table 1.

### Western blot analysis

Western blot analysis was performed as previously described (32, 40). Firstly, the pituitary samples (*n* = 3) were lysed separately in 40 μL of RIPA lysis buffer (Fudebio-Tech, China) containing a protease inhibitor cocktail (1:100, Fudebio-Tech), mixed with 10 μL of 5× DualColor Protein Loading Buffer (Fudebio-Tech). Then the mixture was heated at 100°C for 10 min. Secondly, the lysates and PageRuler^TM^ prestained protein ladders (Thermo Fisher, USA) were separated on 12% SDS-PAGE gels and transferred to PVDF membranes (Pall, USA). Thirdly, the membranes were incubated 30 min in 5% BSA resolved in TBST prior to the incubation of the primary antibody for blocking and cut into pieces according to visible marker bands (25 and 35 KD). The membranes with larger Mw proteins were incubated with GAPDH mouse monoclonal primary antibodies (1:4,000, Proteintech, RRID: AB_2107436), and the ones with smaller Mw proteins were incubated with zebrafish specific FSHb (follicle-stimulating hormones, 1:3,000, RRID: AB_2651068) or LHb (luteinizing hormones, 1:6,000, RRID: AB_2651067) rabbit polyclonal primary antibodies, as previously reported (40). After rinsed with TBST solution for 5 times and 3 min each, they were incubated in peroxidase-conjugated second antibody and lastly visualized with enhanced chemiluminescence (ECL) reagents (Millipore, USA). All Western blot analyses were repeated twice at least.

### Statistical analyses

All data are expressed as mean values ± SEM, and the data were analyzed by Student's *t*-test method with the GraphPad 6.0 software (GraphPad Software, San Diego, CA). *P* < 0.05 was considered statistically significant.

## Results

### Establishment of *esr1* deficient zebrafish line

To disrupt *esr1* gene in zebrafish, TALEN target site for the zebrafish *esr1* gene was designed. There are 9 exons in zebrafish *esr1* gene. The TALEN target site was located on the third exon (Figure 1A). Twenty four hours after injection of paired TALENs, 45.8% (11/24) of embryos sampled carried mutations. The remaining P0 embryos were raised to adulthood and genotyped by sequencing. The mutation rate was 50% (9/18) in the adult P0 fish. The P0 zebrafish are genetically mosaic and the ones carrying germline mutations were crossed with wild-types to produce F1 offspring. An 8-bp deletion mutation (Figure 1A), inducing an open reading frame (ORF) shift (Figure 1B) and early termination of translation (Figure 1C), was chosen to establish the *esr1* mutant line.

### Morphology and ovarian histology of *esr1* mutant female zebrafish

**Figure 2:**
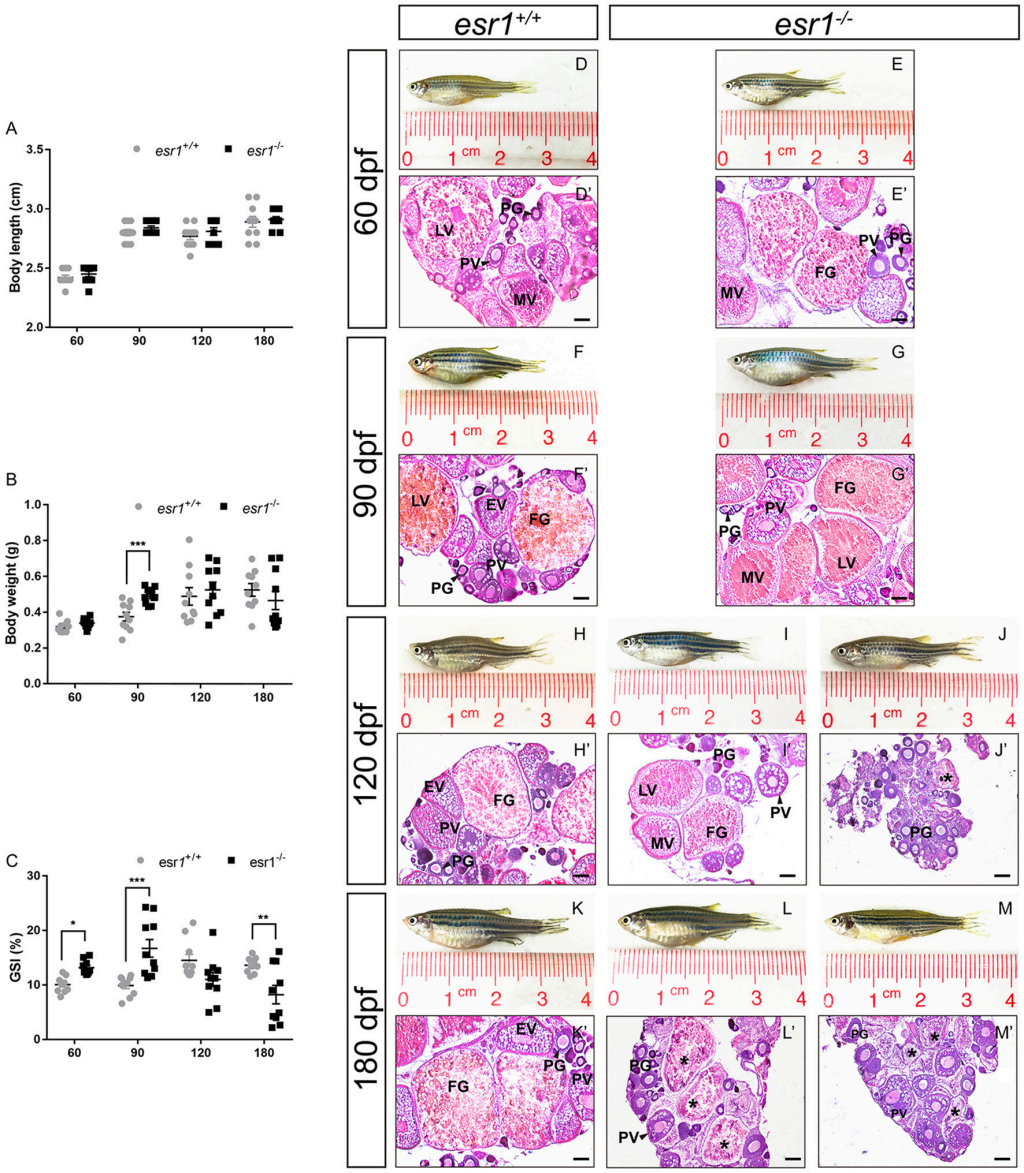
Morphological and histological analyses of *esr1* mutant female zebrafish. **(A)** Body length of *esr1*^+/+^ and *esr1*^−/−^ female zebrafish. **(B)** Body weight of *esr1*^+/+^ and *esr1*^−/−^ female zebrafish. **(C)** GSI of *esr1*^+/+^ and *esr1*^−/−^ female zebrafish. **(D–M)** Gross morphology of female zebrafish. **(D**′**-G**′**)** Ovaries of *esr1*^+/+^ and *esr1* mutant were normal at 60 and 90 dpf. **(H**′**-M**′**)** Part of *esr1* mutant ovaries of at 120 dpf and all *esr1* mutant ovaries were degenerated by the 180 dpf. In these degenerated ovaries, MV and FG follicles were absent and PG follicles, PV follicles and atretic follicles (asterisk) were found. PG, primary growth follicle; PV, pre-vitelligenic follicle; EV, early-vitelligenic follicle; MV, middle-vetelligenic follicle; LV, late-vitelligenic follicle; FG, full grown follicle; ^*^, atretic follicle.

Although the body length of both lines did not show significant difference during lifespan (Figure 2A), the body weight of *esr1* mutant female zebrafish was significantly higher at 90 dpf (Figure 2B). As well, the GSI of *esr1* mutant female zebrafish significantly increased at 60 and 90 dpf but dramatically declined at 180 dpf compared to wild-type zebrafish (Figure 2C).

Morphological and histological analyses were conducted to pubertal (60 dpf) wild-type and *esr1* mutant females. No significant signs of abnormality in the gross morphology of both genotypes of zebrafish were observed (Figures 2D,E). Furthermore, histological analyses showed similar ovarian developmental stage in both zebrafish lines that follicles undergoing primary growth stage (PG), pre-vitellogenic stage (PV), early vitellogenic stage (EV), mid-vitellogenic stage (MV), late vitellogenic stage (LV), and full grown stage (FG) were all found (Figures 2D′,E′). A prolonged observation on the gross morphology and histology of the ovaries was conducted in adult wild-type and *esr1* mutant females from 90 to 180 dpf. The body and the abdomen size of the *esr1* mutant females were indistinguishable with wild-type zebrafish at 90 dpf (Figures 2F,G). But some mutant females with smaller abdomen size were observed at 120 and 180 dpf (Figures 2H–M). Histological analyses showed that similar to wild-type zebrafish, follicles ranged from PG to FG stage were all present in *esr1* mutant ovaries at 90 dpf (Figures 2F′,G′). However, in part of ovaries at 120 dpf and all ovaries at 180 dpf, MV, LV, and FG follicles were absent with only early stage follicles (PG and PV stage follicles) and atretic follicles (Figures 2H′-M′).

### *esr1* mutant female zebrafish developed enhanced fertility but premature ovarian failure

**Figure 3:**
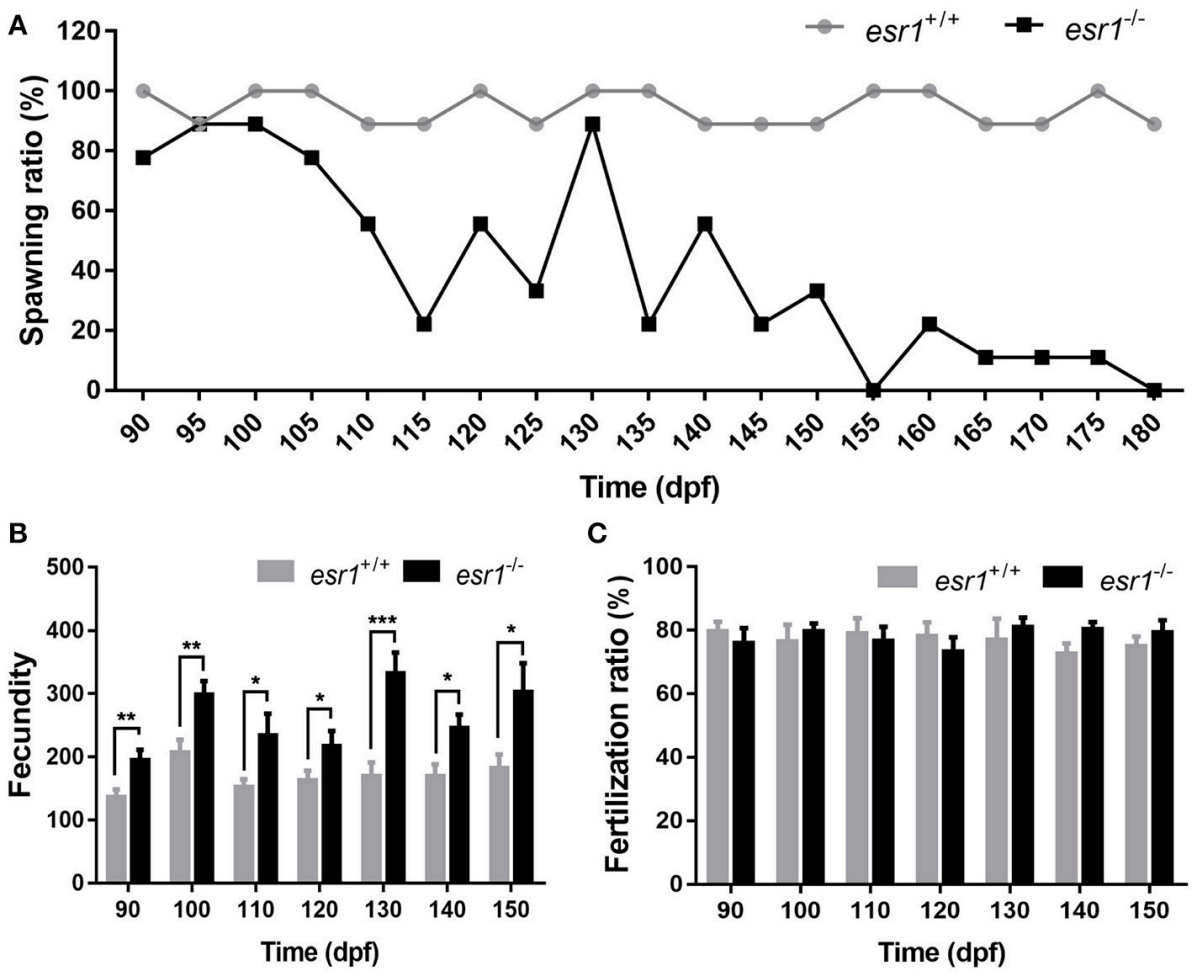
Tracing assessment of fertility on *esr1* mutant female zebrafish. **(A)** Spawning rate of *esr1* mutant female zebrafish was normal at 90 dpf but violently declined at 180 dpf. **(B)** Fecundity was significantly higher in *esr1* mutant female zebrafish. **(C)** Fertilization rate was normal in *esr1* mutant female zebrafish. (^*^P < 0.05; ^**^P < 0.01; ^***^P < 0.001; *n* = 10; Mean ± SEM).

Tracing assessment of fertility was conducted to both wild-type and *esr1* mutant females by pairing them with wild-type males. As illustrated, *esr1* mutant female zebrafish possessed normal fertility as wild-type females at 90 dpf. However, they showed signs of an age-dependent fertility loss from 105 dpf that the number of spawned *esr1* mutant zebrafish reduced with age. By 180 dpf, all *esr1* mutant females were infertile, while the age-matched wild-type females were still fertile (Figure 3A). Moreover, *esr1* deficient zebrafish produced more eggs compared to age-matched wild-type zebrafish from 90 to 150 dpf (Figure 3B). Nevertheless, disruption of *esr1* did not affect the fertilization rate in zebrafish (Figure 3C).

### Vitellogenesis in *esr1* mutant female zebrafish

**Figure 4:**
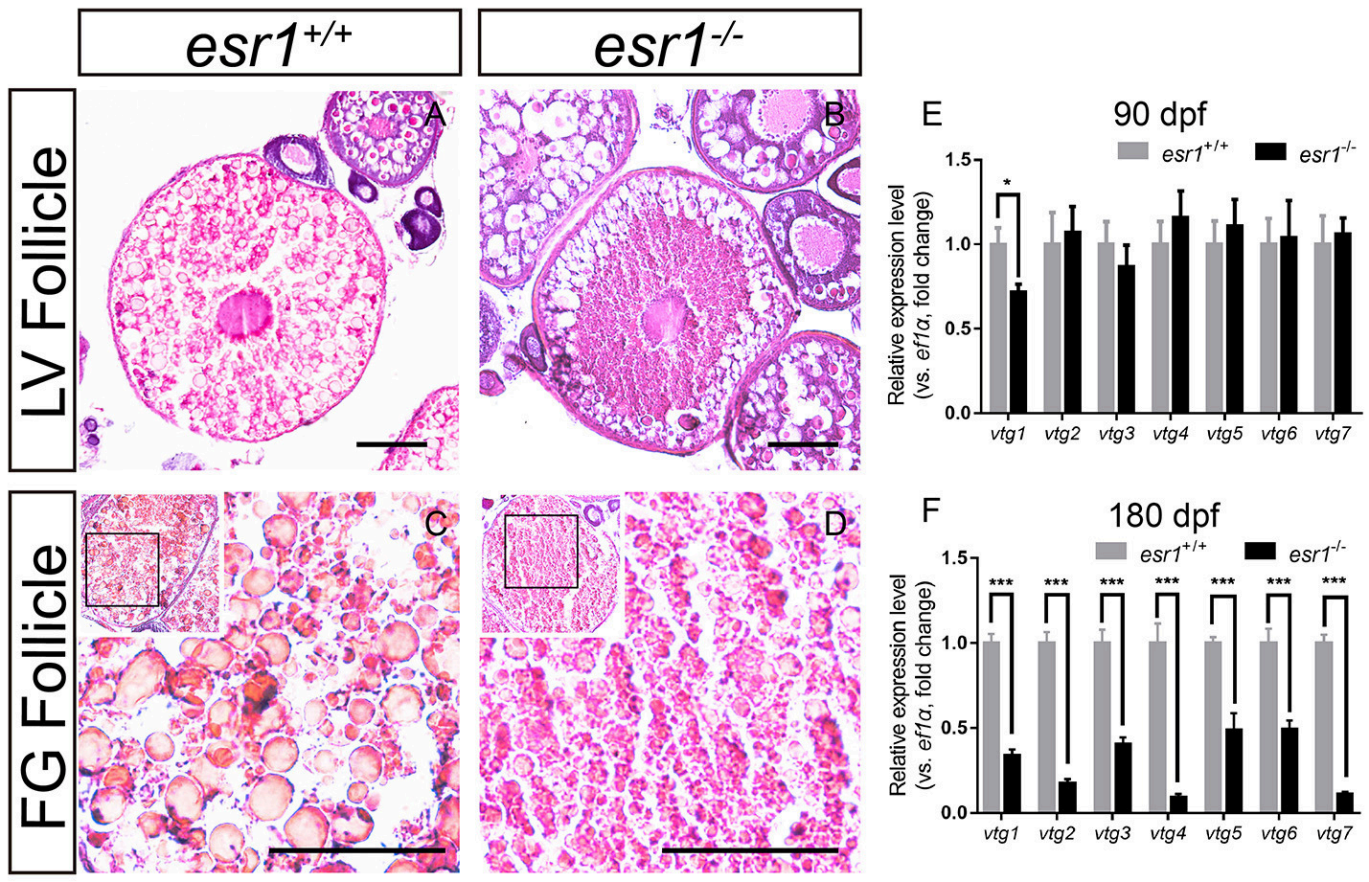
Vitellogenesis in *esr1* deficient female zebrafish. **(A)** LV stage follicles were filled with vitellogenic granules in wild-type zebrafish. **(B)** Vitellogenic granules were found in the lateral part of LV stage follicles in *esr1* mutants. **(C)** FG stage follicles were filled with big full vitellogenic granules in wild-types. **(D)** Only small vitellogenic granules were found in FG stage follicles of *esr1* mutants. **(E,F)** Expression level of seven *vtg* genes at 90 dpf **(E)** and 180 dpf **(F)**. The mRNA levels were normalized to *ef1a*. Average mRNA levels in ovaries of wild-types were defined as 1.0. (^*^P < 0.05; ^***^P < 0.001; Mean ± SEM).

It was noted that a majority number of LV stage follicles in *esr1* mutant female ovaries were abnormal morphologically. LV stage follicles of wild-type zebrafish were filled with vitellogenic granules (Figure 4A). However, vitellogenic granules were only found in the lateral part of *esr1* mutant LV stage follicles (Figure 4B). Similarly, FG stage follicles were filled with large size vitellogenic granules in wild-type follicles (Figure 4C), while few large vitellogenic granules were found in *esr1* mutant FG stage follicles (Figure 4D). To further analyze the molecular mechanism underlying, the expression levels of *vtg* genes in liver were evaluated. The expression level of *vtg1* was significantly decreased at 90 dpf in spite of the other six *vtg* genes (Figure 4E). However, all *vtg* genes decreased violently in the liver by the age of 180 dpf (Figure 4F).

### Steroidogenesis pathway in *esr1* mutant female zebrafish

**Figure 5:**
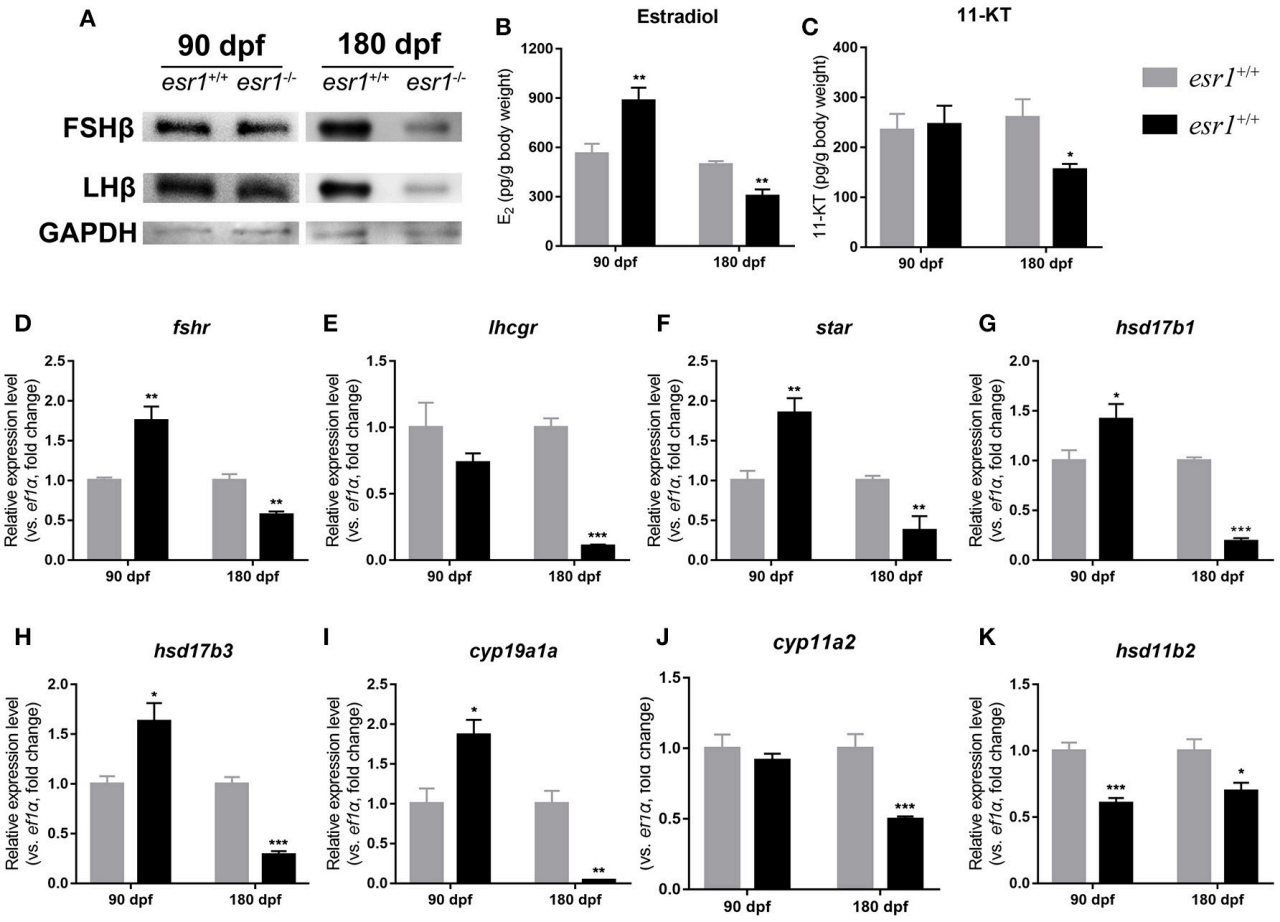
Steroidogenesis in *esr1* mutant female zebrafish. **(A)** FSHb and LHb levels at 90 dpf and 180 dpf were measured via Western bolt analysis. GAPDH was used as the reference. **(B,C)** Whole-body estradiol **(B)** and 11-KT **(C)** levels were measured via ELISA. **(D–K)** The relative expression levels of steroidogenesis pathway genes in the ovaries of wild-type and *esr1* mutant zebrafish at 90 dpf and 180 dpf. The mRNA levels were normalized to *ef1a*. Average mRNA levels in the ovaries of wild-type were defined as 1.0. (^*^P < 0.05; ^**^P < 0.01; ^***^P < 0.001; Mean ± SEM).

Western blot analyses showed that the pituitary protein levels of both FSHb and LHb remained unchanged in *esr1* mutant zebrafish at 90 dpf, yet they both significantly declined by the age of 180 dpf (Figure 5A). Then, E_2_ and 11-KT levels in whole body were measured. E_2_ level was significantly higher in *esr1* mutant females at 90 dpf, while significantly lower at 180 dpf (Figure 5B). The 11-KT concentration was unchanged at 90 dpf, but significantly decreased at 180 dpf in *esr1* mutant females (Figure 5C). Furthermore, panels of genes involved in steroidogenesis in the ovary were analyzed. At 90 dpf, expression levels of genes in steroidogenesis pathway were significantly up-regulated in *esr1* mutant females, including *star, hsd17b1, hsd17b3, cyp19a1a*, and *fshr* (Figures 5D,F–I). Expression of *lhcgr* and *cyp11a2* mRNA levels were unchanged (Figures 5E,J) and the expression level of *hsd11b2* decreased significantly at this age (Figure 5K). However, the expression of all these genes significantly decreased in *esr1* mutant females at 180 dpf (Figures 5D–K).

### IGF system and mTOR signaling pathway in *esr1* mutant female zebrafish

**Figure 6:**
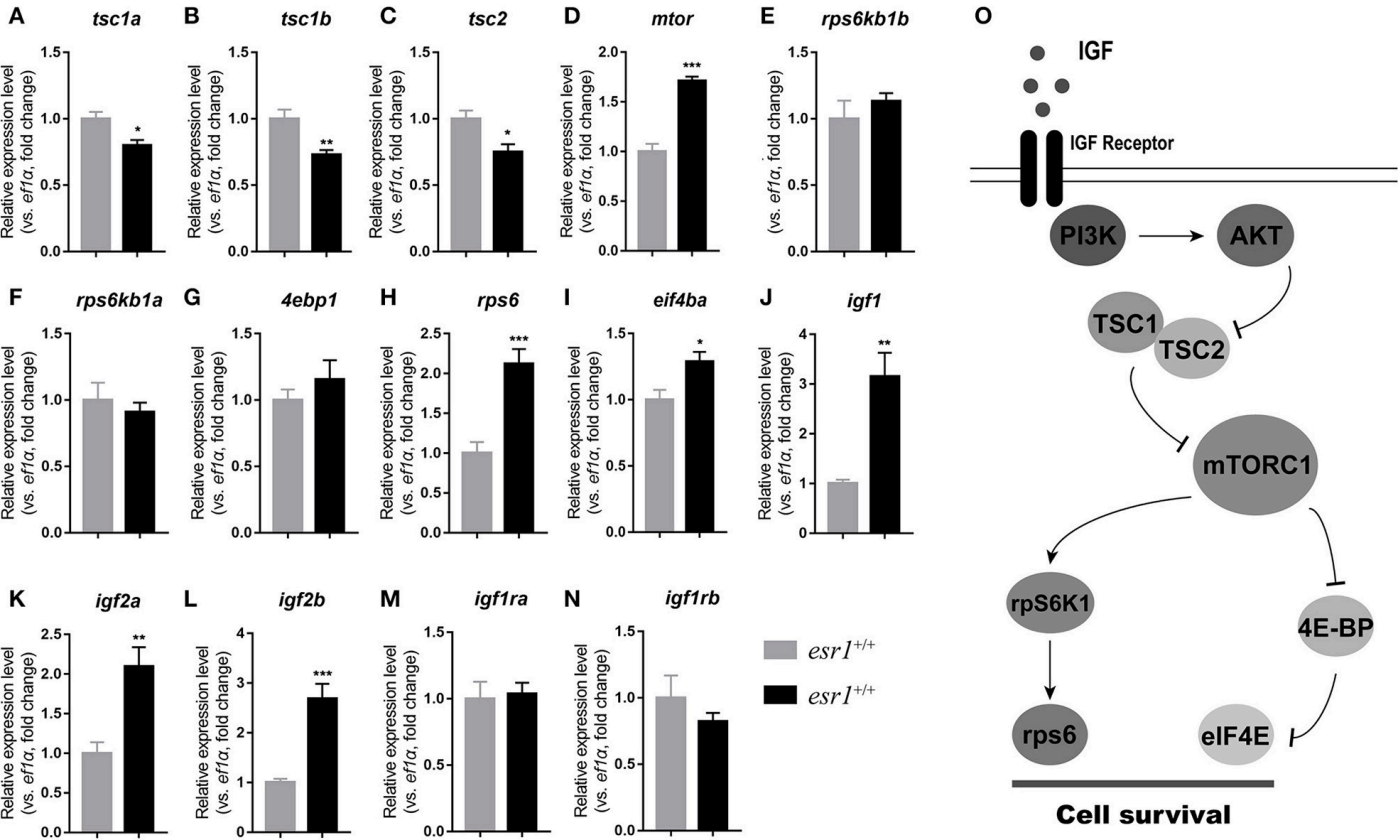
IGF system and mTOR signaling pathway in *esr1* mutant female zebrafish. **(A–I)** The relative expression levels of mTOR signaling pathway genes in the ovaries of wild-type and *esr1* mutant zebrafish at 90 and 180 dpf. **(J–N)** The relative expression levels of *igf* /*igfr* genes in the ovaries of wild-type and *esr1* mutant zebrafish at 90 and 180 dpf. The mRNA levels were normalized to *ef1a*. Average mRNA levels in the ovaries of wild-type were defined as 1.0. (^*^P < 0.05; ^**^P < 0.01; ^***^P < 0.001; Mean ± SEM). **(O)** Diagrammatic sketch of mTOR signaling pathway.

At 90 dpf, the transcriptional levels of *tsc* genes were significantly down-regulated (Figures 6A–C). Expression of *mtor* significantly increased in *esr1* mutant female zebrafish (Figure 6D). The downstream transcriptional factors including ribosomal protein S6 kinase b (*rps6kb1a* and *rps6kb1b*) and eukaryotic translation initiation factor 4e binding protein 1 (*4ebp1*) were unchanged (Figures 6E–G). The mRNA levels of eukaryotic translation initiation factor 4e (*eif4ea*) and ribosomal protein S6 (*rps6*) significantly elevated in *esr1* mutant females (Figures 6H,I). Furthermore, *igf1, igf2a*, and *igf2b* were significantly up-regulated compared to wild-type females (Figures 6J–L). But the expression levels of two *igf1r* genes (*igfr1a* and *igfr1b*) did not show significant difference between wild-type and *esr1* mutant females (Figures 6M,N). However, by the age of 180 dpf, the expression levels of both mTOR signaling pathway and the IGF system were comprehensively down-regulated (Figures 6A–N). Diagrammatic sketch of mTOR signaling pathway was presented in Figure 6O.

### Inhibition of mTOR signaling pathway led to ovarian failure

**Figure 7:**
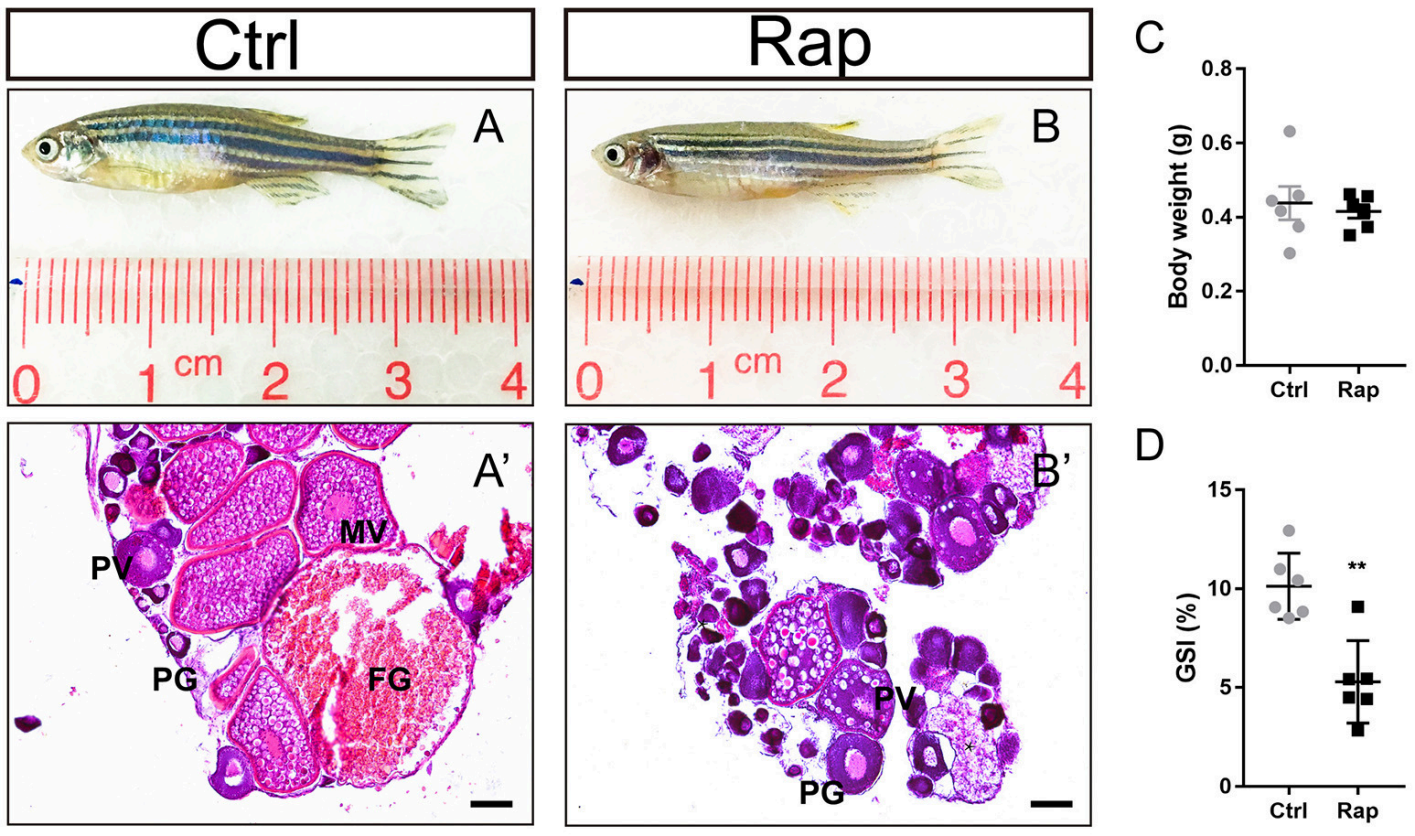
Morphology and ovarian histology of rapamycin treated zebrafish **(A,B)** Gross morphology of control and rapamycin treated zebrafish. **(A**′**,B**′**)** In rapamycin treated zebrafish, only PG follicles, PV follicles and atretic follicles (asterisk) were found in the ovaries. **(C,D)** Body weight **(C,D)** GSI of control and rapamycin treated zebrafish. (^*^P < 0.05; ^**^P < 0.01; ^***^P < 0.001; Mean ± SEM). PG, primary growth follicle; PV, pre-vitelligenic follicle; ^*^, atretic follicle.

In order to clarify the role of mTOR signaling pathway in ovarian maintenance, *in vivo* exposure to rapamycin, an mTOR inhibitor, was conducted to adult wild-type female zebrafish. After treatment with 50 nM rapamycin for 14 days, the gross morphology (Figures 7A,B) and the body weight (Figure 7C) of zebrafish were unchanged, yet these zebrafish were infertile. In this case, the GSI of the rapamycin treatment group was significantly lower compared to the control group (Figure 7D). Moreover, MV, LV, and FG stage follicles were absent in ovaries of rapamycin treated zebrafish. Only PG and PV stage follicles and a few atretic follicles were found, while the ovaries in control group were normal (Figures 7A′,B′).

## Discussion

ERα was shown to be significant in female reproduction in human and mice models. Evidence in teleost fish also suggested its significance in female reproduction. Thus *esr1* mutant zebrafish line was generated to investigate the function of *esr1* in fish ovary. In mammals, global knockout of *Esr1* in mice resulted in infertility with primary amenorrhea (12, 19). Whereas, conditional knockout of *Esr1* in several positions of HPG axis caused milder degree of fertility impairment [Table 1; (7, 23, 24)]. In zebrafish, ERα is the unique alpha subtype of estrogen receptor. The expression of *esr1* is detected in ovary of zebrafish. However, its function in ovary remains unclarified. In a recent work, an *esr1* mutant line of zebrafish was generated via CRISPR/Cas9 system. Sex differentiation, gonad development and reproductive function were normal in *esr1* deficient zebrafish. Fertility assessment found that they were fertile by the age of 120 dpf (Table 1). However, double knockout of ERβs (*esr2a*^−/−^; *esr2b*^−/−^) resulted in completely arrested follicle development and female-to-male sex reversal, suggesting the significance of ERβs in maintenance of female sex status in zebrafish (42). In addition, disruption of *esr1* in medaka did not cause defects in gonadal development or sexual characteristics, which is similar to the phenotype in zebrafish [Table 1; (41)]. In the present study, we found that the *esr1* deficient zebrafish produced more eggs with normal ovarian histology at 90 dpf. But the number of fertile females decreased with age. By the age of 180 dpf, *esr1* mutant female zebrafish developed POF with highly degenerated ovaries, which is consistent with the phenotype in a theca cell specific *Esr1* knockout mice model (7). In zebrafish suffering from POF, MV, LV, and FG follicles were absent and only PG, PV and atretic follicles were found in their ovaries. Similarly, theca cell specific *Esr1* knockout mice over 4-month age possessed fewer corpora lutea but more antral follicles in their ovaries than the age-matche wild-type mice (7). The female reproductive phenotypes of rodent and teleost models were summarized in Table 1, and the similarities between mice and teleosts suggest that the function of ERα in ovary may be significant and conserved in vertebrates.

**Table 1:** Summary of female reproductive phenotypes of rodent and teleost models.

**Species**	**Genotype**		**Fertility**	**Ovary**	**LH**	**FSH**	**E2**	**T/11-KT**	**References**
Mice	^*^G-Esr1 KO		Infertile	Absent ^*^CL	High	Normal	High	High	(12, 19, 22)
	^*^N-Esr1 KO		Infertile	Absent CL	^*^ND	ND	ND	ND	(23)
	^*^Pit-Esr1 KO		Subfertile/Infertile	Reduced/Absent CL	High	ND	High	ND	(24)
	^*^Th-Esr1 KO	4 months	Fertile	Normal	Low	Normal	ND	High	(7)
		6 months	Infertile	^*^POF	Low	Normal	ND	High	
Medaka	G-Esr1 KO		Fertile	Normal	ND	ND	ND	ND	(41)
Zebrafish	G-Esr1 KO		Fertile	Normal	ND	ND	ND	ND	(42)
	G-Esr1 KO	90 dpf	Fertile	Normal	Normal	Normal	High	Norma	Current study
		180 dpf	Infertile	POF	Low	Low	Low	Low	

* *G-, global; N-, neuron; Pit-, pituitary; Th-, theca cell; CL, corpora lutea; POF, premature ovarian failure; ND, not determined*.

The expression levels of vitellogenin (*vtg*) genes were down-regulated in *esr1* mutant zebrafish. Similarly, the estrogenic effect on vtg in *esr1* knockout medaka was significantly attenuated (41). To date, at least seven *vtg* genes (*vtg1–7*) are identified in zebrafish, encoding heterogeneous vitellogenins with three distinct types of Vtgs: type I (Vtg1, 4–7), type II (Vtg2), and type III (Vtg3). All seven v*tgs* are predominantly expressed in female liver, among which *vtg1* is the highest expressed one and type I Vtg is considered to be the predominant form in zebrafish (43–46). As demonstrated in the present work, *vtg1* was suppressed as a result of *esr1* deletion regardless of the other six *vtg* genes, which may be a cause for the histological alteration in *esr1* mutant zebrafish follicles. However, work on protein level is warranted to draw a conclusion.

In teleosts, steroid hormones are deeply involved in the regulation of folliculogenesis (47). Synthesis of steroid hormones is under the control of gonadotropins (38, 47). As illustrated, the protein levels of FSHb and LHb did not alter significantly at 90 dpf, which differs from the *Esr1* deficient mice models (Table 1). However, the mRNA level of *fshr* increased. As a result, the steroidogenesis pathway was up-regulated. Rather than LH, FSH stimulates follicular growth and steroid hormone production which is mainly mediated by *fshr* (48). *Fshr* has been shown in mammals that it can mediate the effect of FSH through the Pi3k/Akt/mTOR signaling pathway (49). Additionally, the involvement of ERα in FSH feedback regulation is clearly elucidated in mice (24, 50). In zebrafish, both FSH and *fshr* are sensitive to estradiol (48, 51). Therefore, loss of *esr1* caused disorders in FSH and *fshr* regulation, which may up-regulate steroidogenesis pathway and production of estradiol at 90 dpf.

The Pi3k/Akt/mTOR signaling pathway is generally considered to be closely related to POF in mammals. In human, activation of this signaling pathway leads to premature and irreversible initiation of primordial follicle pool, and eventually results in premature follicle depletion and ovarian failure (52). Similarly, conditional knockout of *Tsc1, Tsc2*, and *Pten* specific in mice oocytes leads to up-regulation of mTOR signaling pathway and global activation of primordial follicles prematurely, which eventually causes POF (53–56). In the present work, the expression levels of genes in mTOR signaling pathway were assessed. At 90 dpf, the expression levels of *rps6* and *eif4ea* increased significantly. And the *igf* /*igfr* system, the direct upstream of the PI3K/AKT/mTOR pathway (57–59), was also up-regulated. It has been shown that the IGF system is present and dynamic in the zebrafish ovary, which functions directly or indirectly in follicle development and ovarian maintenance (60, 61). Thus, the up-regulation of IGF system in *esr1* mutant zebrafish may activate the mTOR signaling pathway, and eventually over-activated follicle development in these zebrafish. Taken together, the up-regulation of IGF system and mTOR signaling pathway is probably led by FSH/*fshr* disorders and results in fertility enhancement at 90 dpf.

On the other hand, by the age of 180 dpf, the IGF system and mTOR signaling pathway were comprehensively down-regulated in *esr1* mutant zebrafish, accompanied with ovarian failure. Treatment with rapamycin, an mTOR inhibitor, caused a similar phenotype in adult female zebrafish, indicating that the ovarian failure in *esr1* mutant zebrafish is closely relevant to the down-regulation of mTOR signaling pathway. Moreover, *fshr* deficient female zebrafish showed a complete failure in follicle activation with follicles arrested in PG to PV transition (39), which is highly consistent with our results. Therefore, down-regulation of *fshr* and mTOR signaling pathway in *esr1* deficient zebrafish may be relevant to ovarian failure. In this work, ovary samples were used for analysis and the expression profiles of genes in ovary were related to age and developmental stages. Indeed, using samples with the same age and same developmental stage would provide more precise evidence for understanding the mechanism by which *esr1* works. However, the stage-matched follicles were not easily available in this case. To clarify this mechanism, further studies were warranted.

Similar atretic follicle presented in this work was also reported in environmental toxicology cases. Chronic, dietary exposure to 2,3,7,8-tetrachlorodibenzo-p-dioxin (TCDD) decreased egg production and spawning success in zebrafish. In this case, transition of pre-vitellogenic stage follicles to vitellogenic stage follicles were inhibited, resulting in follicular atresia (62), which is similar to ovaries of *esr1* deficient zebrafish at 180 dpf. Moreover, long-term exposure to bromophenol 2,4,6-tribromophenol (TBP) caused defects in vitellogenesis in zebrafish (63). These endocrine disruption chemicals (EDCs) are shown to comprehensively influence the reproduction system and cause severe physiological disorder. However, the mechanisms are still unclear. Our present work provides new insights that these EDCs may effect through ERα as well as the mTOR signaling pathway.

In summary, using the TALEN-mediate gene edition system, we generated the *esr1* deficient zebrafish line. Female mutant zebrafish obtained enhanced fertility, but developed POF later. Furthermore, the relevance of mTOR signaling pathway to fertility enhancement and POF was demonstrated. Thus, this mutant line could be a new ideal vertebrate model for clinical research in POF. Collectively, this work provides genetic evidence that ERα is critical for ovarian maintenance in zebrafish, which hence furthers our understanding of the function of ERα in vertebrate reproduction.

## Author contributions

YC, LW, JH, YG, and YL conducted all the experiments and analyzed the data. HT, XL, and HL designed the experiments. YC and HT wrote the manuscript.

### Conflict of interest statement

The authors declare that the research was conducted in the absence of any commercial or financial relationships that could be construed as a potential conflict of interest.
