# Supplementary material for: Fertility Enhancement but Premature Ovarian Failure in esr1-Deficient Female Zebrafish
Source: Front Endocrinol (Lausanne). 2018 Sep 24;9:567. doi: 10.3389/fendo.2018.00567 (PMC6165881; doi:10.3389/fendo.2018.00567)
Supplement: Supplementary file 1 [file Table_1.DOCX]

Supplemental Table 1. Primers used in the present study.

| Gene | Sense primer | Antisense primer |
| --- | --- | --- |
| *esr1* | GTCCAGTGTGGTGTCCTCTCAGC | GCGTAGAA。TTACCTTTCAGTATG |
| *vtg1* | TCCATTGCTGAAAACGACAA | TGCATTCAGCACACCTCTCA |
| *vtg2* | CCAGCTCTGCGTGAAGTTGT | TCATGGATGGGCCTGAGGAG |
| *vtg3* | GGTGGTTCTTGGACTTGGTT | CACAGGAGAGGATGGGATTT |
| *vtg4* | TGTCGAGGTCCTGAAGCACT | GAAGCCAGCGCCTGTAAACT |
| *vtg5* | TGCCATCTTGGCTCTGAGGA | GGGTTCAGCCTCAAACAGCA |
| *vtg6* | CTGCCCAGAGCTGTTGTAGC | AGTTGGCAAAGCCTTCCAGT |
| *vtg7* | AATCCCAGCACTGCGTGAAG | GGTGACCAGCATTGCCCATA |
| *fshr* | CTTTAAAGGAGCCGAAGGC | GTAGACTGAAACGGCCATCA |
| *lhcgr* | TGGGCTGCTGTCAAAGCAAA | TCTGTATGTGCGGTCGAGGT |
| *star* | ACCTGTTTTCTGGCTGGGATG | GGGTCCATTCTCAGCCCTTAC |
| *hsd17b1* | GGTTTGCCATTCAACGAGGTCTAC | CTCCAGCTCCTTGTCTCCAGTCT |
| *hsd17b3* | AAATTCTGGCGATTCATTCC | ACACCCATTGGTGGAAACTT |
| *cyp19a1a* | ATGGCACCGCCGACTACTAT | ATGGCACCGCCGACTACTAT |
| *cyp11a2* | TCCTGTTTAAAGCCGAAGGT | CTCCATCTTTCAGCAGGACA |
| *hsd11b2* | AACAGGCAGGGAGAGAAAGA | CTTCTTCACTGCTCCACCAA |
| *tsc1a* | AGAGGCTGTCGTGAACCAGT | GGCTTTCTGCAGTTCTGCCT |
| *tsc1b* | GCTGCAAGCTTCTGAGAGCA | ATGGCACCGCCGACTACTAT |
| *tsc2* | TCTTCGGCAATGAGGCCAAC | ATGAGTGTCGTACGGAGGCA |
| *mtor* | TGACTGGCTGGAGTGGTTGA | GCTCGGACCAGCACGATAAG |
| *rps6kb1a* | ACCGTACCTCACCCAAGAGG | TGCCAGCAGATCATCCCAGT |
| *rps6kb1b* | TGTCGACTGGTGGAGTCTGG | CAAGCCGAGATGAGGCACTT |
| *4ebp1* | TCCAACCACCAGACGAGTCA | CAATGGAGAGCTGCGACAGT |
| *rps6* | CTCCTCCTCAGCAAGGGTCA | ATCCTGCTAGCCCTCTTGGG |
| *eif4ba* | CTCCCGAGGGAGCCAAGTAA | GGTCTTGCCCTCCAGTCTGA |
| *igf1* | ATGGCACCGCCGACTACTAT | ATGGCACCGCCGACTACTAT |
| *igf2a* | GGTGAAGTCGGAGCGAGATG | ACTTTCGGGCCAGCAGAATG |
| *igf2b* | ATGGCACCGCCGACTACTAT | ATGGCACCGCCGACTACTAT |
| *igf1ra* | ATGGCACCGCCGACTACTAT | ATGGCACCGCCGACTACTAT |
| *igf1rb* | ATGGCACCGCCGACTACTAT | ATGGCACCGCCGACTACTAT |
| *ef1α* | TGGACACAGAGACTTCATCA | AGATACCAGCCTCAAACTCA |
